# Predictive value of biochemical and hematological markers in prognosis of asphyxic infants

**DOI:** 10.22088/cjim.11.4.377

**Published:** 2020

**Authors:** Hassan Boskabadi, Maryam Zakerihamidi, Ali Moradi

**Affiliations:** 1 Department of Pediatrics, Faculty of Medicine, Mashhad University of Medical Sciences, Mashhad, Iran; 2 Department of Midwifery, School of Medicine, Islamic Azad University, Tonekabon, Iran; 3 Orthopedic Research Center, Ghaem Hospital, Mashhad University of Medical Sciences, Mashhad, Iran

**Keywords:** Asphyxia, Neonates, Prediction, Nucleated red blood cells, Interleukin-6, interleukin-1β, Developmental delay, Death, Pro-oxidant/antioxidant balance, Hypoxic ischemic encephalopathy

## Abstract

**Background:**

Asphyxia is one of the main causes of infant mortality and long-term neurologic complications. This cohort study was aimed to compare the diagnostic value of the hematologic and biochemical factors in the prediction of prognosis of asphyxia according to the high prevalence of asphyxia and its complications.

**Methods:**

In this cohort with a two-year follow-up study with availability sampling, 196 term asphyxic infants were involved from 2009 to 2018. A researcher-designed questionnaire was used as the data collection tool containing infantile and maternal particulars as well as the clinical and laboratory assessments. Serum levels of interleukin-1β(IL-1β), IL-6, pro-oxidant/antioxidant balance (PAB), heat shock protein (HSP) and nucleated red blood cells (NRBC) were checked in infants with perinatal asphyxia. Denver II developmental screening test (DDST-II) was performed at 6, 12, 18, and 24 month post-discharge follow-up visits. Data analysis for comparison of infants with normal and abnormal outcomes was performed using student t- test, chi-square, ROC curve, and regression models.

**Results:**

IL-6, IL-1β, PAB, and NRBC count are among the most important predictors of abnormal complications in asphyxic newborns. PAB>22 (HK) showed sensitivity and specificity of 88.6% and 71.6%, respectively in the prediction of complications of asphyxia. The sensitivity and specificity of an IL-6 higher than 28 (pg/mL) in the prediction of complications of asphyxia were found to be 96.1% and 78.6%, respectively. Elevated levels of IL-6 and IL-1β were associated with increased unfavorable outcomes.

**Conclusion:**

Combinations of: IL-1β+ IL-6 + NRBC; IL-6 + HIE grade + PAB; and IL-6+ HIE grade + NRBC had the highest predictive value (100%) for prognosis of asphyxic infants.

In 2-10% of deliveries (1), neonatal asphyxia is a condition caused by failure in placenta and the umbilical cord oxygenation (2). Asphyxia is one of the main causes of infant mortality and/or chronic neurological disabilities in surviving babies (1). Currently, the diagnosis of asphyxia is based on the following findings: Apgar score, atrial blood gases, signs of hypoxic ischemic encephalopathy (HIE), nucleated red blood cells , fetal electronic monitoring during labor, scalp pH along with presence of meconium in amniotic fluid, multisystem involvement during the first 72 hours postpartum, some evidence of acute non-focal cerebral injuries and encephalopathy in early imaging, specific biochemical markers for evaluation of regional cerebral injuries post perinatal asphyxia such as lactate, LDH, adenylate kinase, creatine kinase, cerebral creatine kinase, neuron-specific enolase, interleukins (ILs) (3), S100-β, heat-shock proteins (HSP) (4-6), hematologic markers (the No. of NRBCs in the umbilical cord blood) (7), and oxidant-antioxidant balance (8).

There is no sole indicator for effective prediction of perinatal asphyxia and only a combination of different criteria can be helpful in the diagnosis of perinatal asphyxia (9). Numerous hematologic and chemical biomarkers consisting interlukines (1 & 6 in particular) (3), pro-oxidant/antioxidant balance (PAB) (8), and NRBC have been studied during the last decade for prognosis of perinatal asphyxia. The predictive value of IL-1 has been shown to be higher compared to IL-8 and TNF-α for abnormal neurologic complications in 6 and 12 months after birth (10). Also, infants with abnormal outcomes and severe HIE have shown higher levels of IL-6 compared to those with normal outcomes and moderate or no HIE (11). The oxidant-antioxidant balance changes during stress and asphyxia conditions and has been suggested as a predictive marker in asphyxia prognosis (8, 12, 13). In physiological conditions, a critical balance exists between the formation of free radicals as peroxidants and the restorative antioxidant defense systems that neutralize free radicals. Hypoxic conditions like as birth asphyxia increase free radicals production resulting in peroxide-antioxidant imbalance (14). An NRBC above 450mm (3) with a NRBC/100WBC ratio higher than 3.25 have been reported to have a sensitivity of 90% and specificity of 74.3% in prediction of abnormal neurologic outcomes at 6 months of age (15). The combination of the abovementioned factors (PAB, IL-6, IL1β, NRBC, NRBC/100WBC) in determining the prognosis of asphyxia has not been fully studied in the literature. Despite vast improvements in understanding the physiopathology of asphyxia, there are no certain criteria for the prediction of severity and long-term outcomes of asphyxia. This cohort study was aimed to compare the diagnostic value of the hematologic and biochemical factors in predicting the prognosis of asphyxia according to the high prevalence of asphyxia and its complications.

## Methods

A total of 196 term asphyxic infants who were born in maternity hospital and admitted to neonatal intensive care unit in Ghaem Hospital, Mashhad, Iran, were involved in this two-year follow-up cohort study with availability sampling during 2009 to 2018. Infants with two out of the five following criteria were included in the study: 1) signs of fetal distress heart rate(HR)<100 beat per minute(bpm) with improper fluctuations and late decelerations), 2) thick meconium stained amniotic fluid with hypotonia, HR deceleration, and respiratory depression, 3) 1-min Apgar score<4 and 5-min Apgar score<7, 4) need for resuscitation>1 min with positive pressure ventilation (PPV) and oxygen therapy in the labor room, 5) pH<7.2 or base excess (BE)>-12mEq/L during the first hour after birth. Immaturity, intrauterine growth retardation, congenital and perinatal infections, congenital malformations, hemolytic anemia, and maternal diabetes mellitus or preeclampsia were the exclusion criteria of this study. All phases of this study were supported and funded by the Ethics Committee of Research Department of Mashhad University of Medical Sciences, (IR.MUMS. fm.REC.1395.54, grant number; 941648). Written informed consent was obtained from each infant’s parents.

Blood specimen sampling for IL-1β, IL-6, and PAB assays (ELISA) as well as CBC and peripheral blood smear were performed for all asphyxic infants at the time of admission (16-18). Enzyme linked immunosorbent assay (ELISA) kits (Bender Med system GmbH) were used to measure the serum levels of IL-6 and IL-1β. Serum Hsp 70 antigen concentrations were determined using a sandwich ELISA in-house. The total number of NRBCs as well as the NRBC/100 WBC ratio were measured and calculated through morphologic investigation of the smears^8^. Infants’ clinical and diagnostic examinations, were performed by a neonatology specialist. Infants’ neurologic functions were evaluated on days 1, 3, and 7, and the severity of HIE was defined according to Sarnat grading (19). Hyperconsciousness, irritability, hyperreflexia, and lack of convulsion for at least 24 hours after birth were defined as mild or grade 1 HIE; Lethargy, hypotonia, hyporeflexia, meiotic pupils, and convulsion were defined as moderate or grade 2 HIE; apnea, flaccidity, severe convulsions or coma were defined as severe or grade 3 HIE. Patient evaluations were performed based on clinical and laboratory examinations as well as imaging techniques (chest x-ray, abdominal sonography, and brain CT scan) as per indication. Denver II developmental screening test (DDST-II) was performed at 6, 12, 18, and 24 month post-discharge follow-up visits. DDST-II is a well-known evaluation test for child growth and development from birth to 6 years in 1) personal-social, 2) precise-adaptive movements, 3) language, and 4) coarse movements. Failure in 1, 2, and 3 or more aspects are defined as mild, moderate, and severe developmental retardation respectively (19). In this study any kind of developmental delay or death are defined as abnormal outcome. A researcher-designed questionnaire consisting of maternal and infantile characteristics was used as data collection tool. The infantile characteristics consisted of: 1-min and 5-min Apgar score; weight; IPPV (intermittent positive pressure ventilation) duration; HIE grade; infantile reflexes; cardiopulmonary, head and neck, abdomen, extremities, and muscular tone statuses; convulsion; low consciousness; CT scan results; mechanical ventilation; and laboratory findings. The laboratory findings in the questionnaire were: IL-1β, IL-6, HSP, 1^st^ hour blood gas, No. of NRBCs, NRBC/100 WBC ratio, and PAB.

Statistical Analysis: SPSS 16.5 was used to analyze the data. Descriptive statistic indices including frequency distribution tables, mean and standard deviation were used to describe the characteristics of the study samples. To ensure the homogeneity of the two groups, chi-square test was used for qualitative variables, independent t-test for normal quantitative variables and Mann-Whitney test for non-normal quantitative variables. Regression models were used to determine the predictive values of various indexes in prognosis of asphyxic newborns. In this study, a p<0.05 was considered as significant.

## Results

Thirty five out of 196 infants were excluded from the study due to the parents’ unwillingness for follow-ups (n=15), intrauterine growth restriction (n=3), congenital (n=2) and perinatal (n=3) infections, hemolytic anemia (n=3), congenital malformations (n=2), maternal preeclampsia (n=4) and diabetes (n=3). The total mortality rate among 161 infants was found to be 19.25% (31) infants. Figure 1 depicts the comparison between the asphyxic infants with normal outcome (n=92, 57%) and those who developed abnormal outcome (n=69, 43%). There was a significant statistical difference in sex (P=0.022) and mode of delivery (P=0.009) between the asphyxic infants with normal outcome and those who developed abnormal outcome (fig 2). According to the results of regression models IL-6, IL-1β, and PAB are among the most important defining variables in the prognosis of asphyxic infants. Combinations of “IL-6 + IL-1β + NRBC count”, “IL-6 + PAB +HIE grade”, and “IL-6 +HIE grade + NRBC count” have higher predictive values compared to other combinations in defining the prognosis of asphyxic infants (table 1). According to our findings PAB>22 (HK) has sensitivity and specificity of 88.6% and 71.6%, respectively in the prediction of complications of asphyxia. Also, the sensitivity and specificity of an IL-6 higher than 28 (pg/mL) in the prediction of complications of asphyxia was found to be 96.1% and 78.6%, respectively (figure 3).

The sensitivity and specificity of NRBC>70 in prediction of complications of asphyxia was found to be 80.6% and 76.3%, respectively (figure 2). A significant linear correlation was found between IL-6 and IL-1β with outcomes in asphyxic infants (Sig=0.000 for both, correlation=0.509). Elevated levels of IL-6 and IL-1β were associated with increased unfavorable outcomes. Data analysis based on regression models showed that variables like IL-6, IL-1β, PAB, and NRBC count are among the most important predictors of abnormal complications in asphyxic newborns. 

**Figure1:**
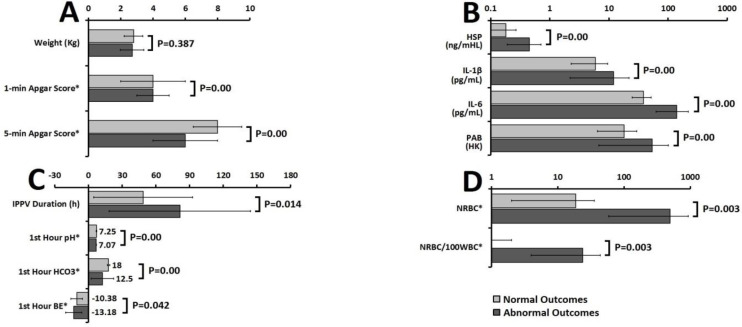
Comparison of early A) clinical, B) serologic, C) ventilatory support and atrial blood gases analysis, and D) hematologic investigations of asphyxic newborns with normal and abnormal outcome

**Figure 1:**
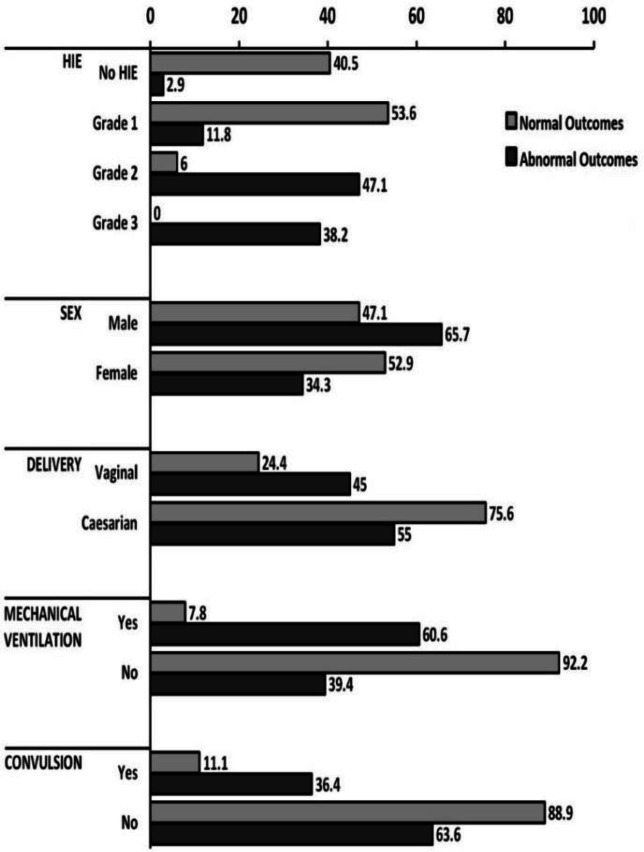
Comparison of maternal and infantile variable in asphyxic newborns with normal and abnormal outcomes

**Table 1:** Predictive value of various combinations of prognostic parameters for complications in asphyxic newborns

**Diagnostic Variable/Combination**	**-2 log likelihood**	**Cox & Snell R** ^2^	**Nagelkerke** **R** ^2^	**Hosmer-Lemeshow Test**	**Correct Predicted Percentage **
IL-6 + HIE grade+ PAB	0.000	0.743	1.000	1.000	100
IL-6 + HIE grade+ NRBC count	0.000	0.742	1.000	1.000	100
IL-6+IL-1β + NRBC count	0.000	0.737	1.000	1.000	100
IL-6+IL-1β + HIE grade	16.431	0.659	0.879	0.140	96.2
IL-6 + HIE grade	21.177	0.648	0.864	0.060	93.5
IL-6+IL-1β	29.638	0.563	0.750	0.000	92.5
IL-6	39.243	0.529	0.705	0.000	91.9

**Figure 3:**
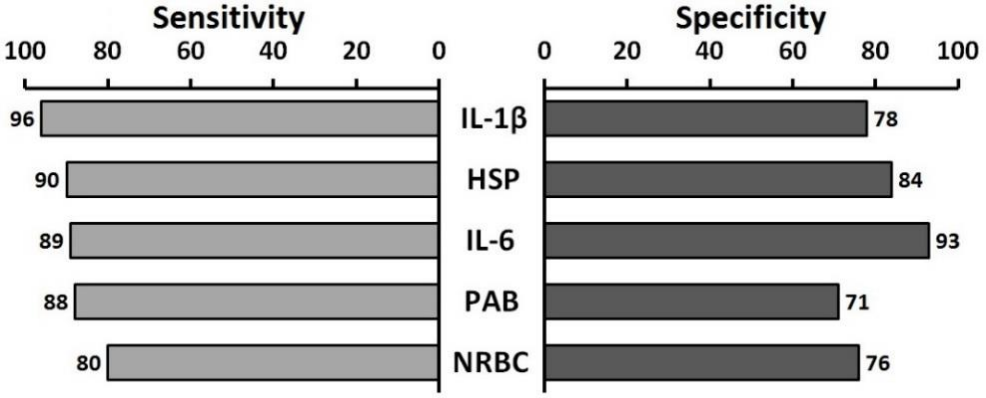
Sensitivity and specificity of laboratory biomarkers in the prediction of prognosis of asphyxia

## Discussion

The results of a two-year follow-up of 161 asphyxic infants showed that 57% of infants had normal outcomes, 20% had passed away, and 23% had abnormal complications among which 45%, 21%, and 34% had mild, moderate, and severe developmental delay, respectively. A similar study has already reported death in ¼ and developmental complications in some more of asphyxic newborns (10). Asphyxic infants with abnormal outcome showed higher PAB compared to those with normal outcomes in our study, which is in line with a previous study showing significantly higher PAB and lower pH in asphyxic infants compared to normal newborns (8). Also the highest PAB ratios were seen in infants with grade 3 HIE (19, 8). study showed PAB-levels in neonates with normal and abnormal outcomes were 17.1±9.23 and 48.27±41.30 HK, respectively. A combination of HIE-intensity and PAB, compared to other indicators, had a higher predictive-value (95.2%) for outcomes in asphyxiated babies (8). HSP appears in cells as a result of impaired cellular function in response to exposure to the environmental stresses. To the best of our knowledge, few studies have reported a significant difference (P=0.001) in HSP levels in asphyxic (0.36 ng/mL) and healthy (0.24 ng/mL) infants (20, 21). HSP level has shown a sensitivity of 90% in prediction of prognosis of asphyxic newborns (20). A significant correlation was found between both NRBC count and NRBC/100WBC ratio; and the final outcomes; moreover, an NRBC count>70 had sensitivity of 70% and specificity of 76.3% in prediction of complications of asphyxia. Asphyxia can affect bone marrow and increase NRBC count. NRBC count has already been known as a marker for hematopoiesis. The relationship between intrauterine hypoxia and hematopoiesis has also been reported in the literature (22-25). NRBCs are immature form of erythrocytes generated and reserved in bone marrow as precursors of mature erythrocytes and reticulocytes in response to erythropoietin. Several acute and chronic stimulators can increase erythropoietic action or trigger sudden release of NRBCs from bone marrow (26). In asphyxic conditions, increased production of erythropoietin results in gradual maturation and release of NRBCs into the blood stream; and hence, presence of NRBCs in the umbilical blood indicates intrauterine asphyxia (22, 27). A sensitivity and a specificity of 85% and 90%, respectively, have been reported for NRBCA count>11/100 leukocytes in the prediction of unfavorable complications of asphyxia (22). Also, NRBC count>70 was reported to have a sensitivity and specificity of 83.4% and 73.5% in the prediction of diagnosis of perinatal asphyxia in a previous study (7). The results of the current study showed that unfavorable outcomes of asphyxia were associated with higher levels of IL-1β and IL-6 as valuable predictive factors. Cerebral hypoxic/ischemic injuries upregulate the expression of cerebral inflammatory cytokines (IL-1β and IL-6) (28). Significantly, higher serum and CSF levels of IL-1β, IL-6, and TNF-α have been reported in infants with HIE. CSF IL-1β level was also reported to be the best predictor for abnormal neurologic complications and the DDST-II at 6 and 12 month after birth (29). Post-perinatal asphyxia CSF levels of IL-6 were correlated with the severity of infantile HIE, cerebral injury, and neurologic complications (11). A systematic review on the issue suggests that IL6 > 41 Pg/dl has the sensitivity of 84.88% and the specificity of 85.43%, whereas IL-1*β* >4.7 Pg/dl has the sensitivity of 78% and specificity of 83% in the diagnosis of neonatal asphyxia (30). Among the diagnostic ILs for neonatal asphyxia, combination of IL6 and IL-1*β*  had the highest sensitivity, that is, 92.9%.A separate study has reported elevated levels of IL-1β and IL-6 in term asphyxic infants. The predictive value of IL-6 for moderate to severe HIE had a sensitivity of 86% and a specificity of 100% (31). We, in a former study, have reported sensitivity and specificity of 80.5% and 81.6%, respectively, for IL-6; and 71% and 89.1%, respectively, for IL-1β, in diagnosis of perinatal asphyxia (3).

Combination of IL-1β + IL-6 + NRBC count, IL-6 + HIE grade + PAB, and IL-6 + HIE grade+ NRBC count had predictive values of 100% in this study. The highest predictive value reported in a previous study for combination of S-100 protein and CK-BB was 83% with a sensitivity of 95%for moderate and severe HIE (8, 32). Long follow-up time with low cooperative parents was the major restriction in the current study.

In conclusion the results of two-year follow-up of 161 asphyxic infants showed 57% with normal outcomes, 23% had developmental retardation (45% mild, 21% moderate and 34% severe). Asphyxic infants with unfavorable outcomes had higher levels of IL-1β, IL-6, PAB,HSP, IPPV duration, NRBC count, and NRBC/100 WBC; and lower 1-min and 5-min Apgar, and 1st hour pH and BE levels. All in all, PAB>22 HK, NRBC count>70, high grade of HIE, low 1st hour pH, IL-1β>3.3 pg/mL, and IL-6>28 pg/mL are predictors of poor prognosis and mortality within 2 years after birth.

## Ethics approval and consent to participate

Written informed consent was obtained from each infant’s parents.
